# Costs and advance directives at the end of life: a case of the ‘Coaching Older Adults and Carers to have their preferences Heard (COACH)’ trial

**DOI:** 10.1186/s12913-015-1201-9

**Published:** 2015-12-09

**Authors:** Billingsley Kaambwa, Julie Ratcliffe, Sandra L. Bradley, Stacey Masters, Owen Davies, Craig Whitehead, Catherine Milte, Ian D. Cameron, Tracey Young, Jason Gordon, Maria Crotty

**Affiliations:** Flinders Health Economics group, Flinders University, A Block, Repatriation General Hospital, 202-16 Daws Road, Daw Park, SA 5041 Adelaide, Australia; CareSearch, Palliative care knowledge network, Palliative and Supportive Services, Flinders University, PO Box 2100, Adelaide, SA 5001 Australia; The Discipline of General Practice, Health Sciences Building, Flinders University, GPO Box 2100, SA 5001 Adelaide, Australia; Department of Rehabilitation and Aged Care, Flinders University, C Block, Repatriation General Hospital, 202-16 Daws Road, Daw Park, SA 5041 Adelaide, Australia; School of Exercise and Nutrition Sciences, Deakin University, Melbourne Burwood Campus, 221 Burwood Highway, Burwood, VIC 3125 Melbourne, Australia; John Walsh Centre for Rehabilitation Research, University of Sydney, Level 13, Kolling Institute of Medical Research, Corner Reserve Road and Westbourne Avenue, Royal North Shore Hospital, St Leonards, NSW 2065 Sydney, Australia; School of Health and Related Research, The University of Sheffield, Regent Court, 30 Regent Street, Sheffield, S1 4DA UK; Discipline of Public Health, University of Adelaide, 178 North Terrace, Terrace Towers, SA 5005 Adelaide, Australia; Billingsley Kaambwa, Flinders Health Economics Group, School of Medicine, Flinders University, Repatriation General Hospital, 202-16 Daws Road, Daw Park, SA 5041 Australia

**Keywords:** End of life, Advance directives, Care plans, Costs

## Abstract

**Background:**

Total costs associated with care for older people nearing the end of life and the cost variations related with end of life care decisions are not well documented in the literature. Healthcare utilisation and associated health care costs for a group of older Australians who entered Transition Care following an acute hospital admission were calculated. Costs were differentiated according to a number of health care decisions and outcomes including advance directives (ADs).

**Methods:**

Study participants were drawn from the Coaching Older Adults and Carers to have their preferences Heard (COACH) trial funded by the Australian National Health and Medical Research Council. Data collected included total health care costs, the type of (and when) ADs were completed and the place of death. Two-step endogenous treatment-regression models were employed to test the relationship between costs and a number of variables including completion of ADs.

**Results:**

The trial recruited 230 older adults with mean age 84 years. At the end of the trial, 53 had died and 80 had completed ADs. Total healthcare costs were higher for younger participants and those who had died. No statistically significant association was found between costs and completion of ADs.

**Conclusion:**

For our frail study population, the completion of ADs did not have an effect on health care utilisation and costs. Further research is needed to substantiate these findings in larger and more diverse clinical cohorts of older people.

**Trial registration:**

This study was registered on 13/12/2007 with the Australian New Zealand Clinical Trial Registry (ACTRN12607000638437).

## Background

Australia has a rapidly ageing population with 14 % being over the age of 65 in 2012 (15 % and 13 % for women and men, respectively) [1, 2] with this figure set to rise to 22 % by 2061 [1]. Older people nearing the end of life are susceptible to multiple comorbid illnesses including neurodegenerative disorders that cause impairments in motor and sensory functioning, mobility and balance as well as chronic conditions such as diabetes, arthritis and cardiovascular disease [3–5]. Many of these comorbidities lead to reduced functional status in older people: chronic disease burden, impaired cognition, diabetes and arthritis all increase the risk of falls [6–10]. Older people aged between 65 and 75 years are twice as likely in South Australia to be admitted to a hospital compared to the general population while those aged over 85 are five times more likely [11]. In addition, approximately 80 % of all deaths in Australia in 2012 were attributable to people aged over 65 years experiencing chronic disease as the leading cause of their death [12, 13]. Further, Australian studies indicate that the majority of older people die in a hospital or another institutional setting and yet this is not the place they would have chosen to die [14].

Many older people nearing the end of life may be unable to make health or other personal decisions due to factors that may inhibit their ability to do so including impaired cognition and decision-making capacity [15]. Substitute decision makers (SDMs), such as family members, carers or healthcare providers, may be required to make decisions for the person at this time and these decisions are often made in an emotional climate [16]. In recent years in Australia, there has been growing interest in end of life care plans or Advance Directives (ADs) to help individuals to make their preferences known when they no longer have an ability to do so [17]. Advance directives are documents that provide the opportunity for people to make instructional decisions and/or nominate SDMs for the future ensuring that their preferences can be known and respected at the time of incapacity [17]. Prior to July 2014, South Australia had four legal ADs: Enduring Power of Attorney (EPA), Enduring Power of Guardianship (EPG), Medical Power of Attorney (MPA) and Anticipatory Direction [18, 19]. Under the Advance Care Directives Act 2013 (SA), the EPG, MPA and Anticipatory Direction were replaced with a new Advance Care Directive form, which was implemented in July 2014. Some non-legal ADs also exist and may form part of an overall advance care plan, such as the Good Palliative Care Plan (GPCP) produced by Palliative Care Australia [20] and the Statement of Choices document (SOC) which is part of the Respecting Patient Choices Program [21]. Non-legal ADs are usually care plans created for a person during a process whereby individuals in consultation with health care providers and relatives, describe their personal values and life goals and these help inform healthcare and lifestyle decisions at times of future health care need [22]. Documenting ADs enables alignment of decision-making to support personal autonomy of individuals with decision-making by families and other decision-makers and has been demonstrated to increase satisfaction with care [23–25].

A number of international studies have documented the relationship between ADs and healthcare costs [26–38]**.** And while some generic end-of-life-care literature [35, 39] suggests a pattern of higher health care expenditures and service utilisation for older people nearing the end of life relative to costs earlier in life, research specifically documenting the impact of ADs on healthcare costs indicates that use of ADs is either associated with lower healthcare costs [26, 29, 32, 36–38] or does not have an impact on these costs [27, 28, 30, 31, 33–35]. Explanations for these results vary. In a study of consecutive American patients who died in a university tertiary care medical centre, Weeks et al. [29] for instance suggested that lower costs for decedents with a previously completed AD could have been due to this group preferring to limit their care while Nicholas et al. [32], based on a study of decedent Medicare beneficiaries, found that such treatment-limiting ADs only had a significant impact in high spending regions characterised by aggressive end-of-life-care. Tan and Jatoi [30], drawing on a sample of cancer patients, concluded that those with ADs had no significant difference in cost from those patients without ADs because a substantial proportion of these ADs lacked specifics about end of life care and therefore did not lead to significant changes in patient care. A similar result was found in the ‘Study to Understand Prognoses and Preferences for Outcomes and Risks of Treatments’ (SUPPORT) trial and Teno and colleagues et al. [31] suggested that this lack of association, and in particular that of ADs not being associated with decreased costs, was reassuring as the “…primary purpose of ADs is to convey patient preferences and instructions to guide future care during a period of mental incapacity, not to save scarce societal resources.” However, little research has been conducted to assess actual health care utilisation and health care expenditures within an Australian setting for older people nearing the end of life who have used ADs. This paper reports on research designed to examine the relationship between healthcare costs and completed ADs whilst controlling for the effect of other factors such as health-related quality of life, demographic characteristics, mortality and place of death. In this study, we also instrument or control for the decision to complete ADs as some factors such as age, gender and quality of life (QoL) have been shown to influence this decision [19, 40–42]. To our knowledge, this is the first time that this type of analysis has been completed for a cohort of frail older people in Australia. The results of this study will be helpful to policy makers and others with regard to the total costs associated with care for older people nearing the end of life and the cost variations related with end of life care decisions.

## Methods

This study was approved by the Research Ethics Committee at the Repatriation General Hospital in Adelaide (approval no. 90/07) and written informed consent was obtained from all participants.

### Data source

Data for this secondary study were obtained from the Coaching Older Adults and Carers to have their preferences Heard (COACH) randomised controlled trial, the methodological details of which have been reported elsewhere [43]. Briefly, the aim of the primary study (the COACH trial) was to determine whether a coaching intervention delivered by a geriatrician and specialist nurse in a post hospital (intermediate) care setting improved older adults’ and carers’ assessment of the quality of preparation for transfers. This intervention included questions on completion of ADs including the GPCP, EPGs and other non-GPCP and non-EPG medical documents (MDs). A total of 230 older adults admitted to Transition Care (a type of intermediate care) in Adelaide, Australia were randomised into the intervention (116) or usual care (114) arms. To be eligible, participants needed to be identified from consecutive admissions to a single residential Transition Care (TC) facility, able to communicate in English and nominate an informal carer willing to participate in the study. The Australian TC Program provides support for older people at the conclusion of a hospital stay and can be provided in a residential or community setting [44]. All participants received usual care, delivered by a multidisciplinary team at a TC facility, made up of comprehensive geriatric assessment, care plans and periodic review at regular case conference meetings [43, 45]. A comprehensive geriatric assessment was carried out in both arms of the COACH trial and included the use of the interRAI Post-Acute Care Assessment instrument [46] as well as a question about the existence of legal documents including ADs. If a participant was identified as palliative and had not completed an AD, TC nurses initiated a discussion with the older person about their preferences for end of life care and a geriatric registrar completed the consultation and signing of the GPCP.

Participants allocated to the intervention group received a coaching intervention that comprised four additional components: a question prompt list used a week before a geriatric consultation in order to enhance patient participation during the consultation; geriatric consultations with a geriatrician and nurse covering several topics included on a checklist; a written summary of every consultation; and a follow-up telephone call two to three weeks post discharge [43]. The question prompt list included a prompt about EPAs and EPGs while the checklist for the geriatric consultation included a prompt to discuss end of life care plans and consider completion of a GPCP.

There are several different types of legal and non-legal ADs including the GPCP (non-legal) and EPG (legal) that were documented in this study. The GPCP documents a person’s preferred medical treatment to be pursued once they have been designated palliative while the EPG is a document which appoints and instructs a substitute decision-maker (e.g. friend or family member) to make decisions concerning the person’s health, residential and lifestyle care should the person be unable to make these decisions [47]. There were also a number of other MDs comprising both legal and non-legal ADs (e.g. Anticipatory Directions, Medical Power of Attorney, Living Wills) that patients completed which either specified the types of medical treatment the person did or did not want at times of decision-making incapacity or nominated a substitute decision-maker [47]. Information on whether these ADs were completed before or during the trial was also collected. This study documents the last ADs that were in place for study participants and focused on the categories of GPCPs, EPGs or MDs (non-GPCP, non-EPG).

### Self-reported patient outcome measures

These measures were collected at baseline, 3 and 12 months and included QoL as measured by the EuroQoL EQ-5D 3 level (EQ-5D 3 L) instrument [48] and functional status assessed using the modified Barthel Index [49]. The difference in EQ-5D 3 L scores between baseline and 12 months was used to generate a ‘QoL change’ variable. Geriatric Depression Scale (GDS‐15) scores for measuring depressive symptoms [50] were also gathered at baseline and 12 months and mortality data were collected at 3 and 12 months. Other information was collected at baseline only and this included data on cognitive status as assessed by the Standardised Mini Mental State Examination (SMMSE) [51] and body mass index (BMI). On discharge from TC, information on whether, the type and when the ADs had been completed was extracted from the TC facility records.

### Cost analysis

Costs were analysed from a health sector perspective. Within the COACH trial, Medicare costs for out-of-hospital visits were estimated using the Medicare Benefits Scheme (MBS) public subsidy (the Medicare benefit) paid for each of these items. Medicare costs for out-of-hospital visits included costs for tests, treatments and investigations; medical consultations and costs for primary care and general practice visits (Other). For in-patient hospital admissions, data on the diagnosis related group (DRG) associated with each admission as well as the length of stay were collected from hospitals. Using the Australian Refined Groups DRG cost weights [52], a cost per day for each DRG was established and this was multiplied by patient level data capturing the length of stay for each DRG-associated admission within the COACH data in order to obtain a total cost for in-hospital admissions. Medicare drug costs were estimated using the Pharmaceutical Benefits Scheme (PBS) public subsidy (the Pharmaceutical benefit) paid for each of the drugs used. Resource use data collected for the intervention included staff time spent by geriatricians and nurses, equipment, overheads and other supplies used. Salary costs for staff, unit costs for equipment, overheads and supplies were obtained locally from hospital finance departments.

All costs were reported in Australian dollars at 2013/14 unit prices and discounting was not necessary as the follow up period was less than one year [53].

### Statistical analysis

Descriptive statistics (means and standard errors) were generated and simple statistical tests of differences that took into account the distributional nature of the variables in the dataset (Kruskall Wallis and Pearson chi-square tests) were also carried out [54]. Differences in demographic and other participant characteristics according to whether or not ADs were completed were tested. A two-step endogenous treatment-regression approach was used in our analysis [55]. In the first step, the decision to complete an AD was predicted from a number of factors identified in the literature [19, 40–42] including age, gender and quality of life status using a probit regression model. This was because we assumed that AD completion was endogenous with the decision to complete/not complete ADs ultimately affecting healthcare costs. Not accounting for this endogeneity, if present, leads to biased estimates related to Type I and Type II errors [55, 56]. In the second step, a linear regression model was utilised to ascertain whether a number of independent variables (including the hazard or probability of completing ADs obtained from the first step) were jointly predictive of lower or higher health care costs on average [55]. The endogenous treatment-regression approach allowed the modelling of a specific correlation structure between the unobservables that affect the decision to complete ADs and those that affect variation in healthcare costs [55–58]. Because our analysis distinguished between seven types of healthcare costs (i.e. costs for drugs; hospitalisation, tests, treatments and investigations; consultations, general practice visits and total costs (unadjusted and adjusted for censoring), we fitted seven two-step regression models using the ‘etregress’ command in Stata [59]. In line with standard practice in analyses such as ours, independent variables included in each final model were chosen on theoretical grounds (i.e. documented or postulated relationships with the dependent variable) as well as on the basis of their ability to improve the model fit [60]. As there were missing data on some costs and other patient variables (Table 1), multiple imputation [61] was used to account for missing values prior to examining the relationship between costs and other patient characteristics. The imputation procedure used an iterative Markov chain Monte Carlo method based on a multivariate normal regression [62] and involved replacing each missing value in the dataset with a set of 20 plausible values that represented the uncertainty about the right value to impute [61]. The 20 resultant multiply imputed datasets were then analysed using standard complete-case procedures and the results combined using Rubin’s rules [63]. The regressions for total costs was also later adjusted for censoring using Lin’s method [64]. A significance level threshold of 5 % (0.05) was assumed as the criterion for determining statistical significance in all analyses [65]. All analyses were conducted in Stata version 13.1 [59].

**Table 1:** Baseline characteristics by whether participants had advance directive completions

Variable	AD^a^ completed	AD^a^ not completed	Entire sample
	*n* = 76	*n* = 152	*n* = 228
	mean (SD)	mean (SD)	mean (SD)
Age (in years)*	85.76 (7.02)	83.31 (6.88)	84.13 (7.01)
Baseline QoL^b^ (EQ-5D 3 L score)*	0.41 (0.31)	0.49 (0.29)	0.46 (0.30)
Baseline functional status (Barthel Index)	57.46 (21.4)	60.99 (20.70)	59.81 (20.95)
Baseline cognitive function SMMSE^c^ score)	22.24 (5.54)	23.63 (4.97)	23.17 (5.22)
Baseline depressive symptoms (GDS-15^d^ score)*	5.84 (2.97)	4.30 (2.97)	4.80 (3.05)
Body mass index (BMI)^e^	24.03 (5.79)	24.47 (5.86)	24.32 (5.83)
	**n (%)**	**n (%)**	**n (%)**
Gender (female)	44 (58)	94 (62)	138 (61)
Individuals with a QoL^f^ gain over 12 months	28 (42)	67 (48)	95 (46)
Individuals dead at 12 months	18 (24)	35 (23)	53 (23)
Place of death			
Hospital	10 (56)	21 (60)	31 (58)
Residential Care/Respite	8 (44)	11 (31)	19 (36)
Home/Independent	0 (0)	3 (9)	3 (6)
Living unit			
Type of AD^a^ completed (%)			
GPCP^g^	35 (46)		
EPG^h^	21 (28)		
MD^i^	20 (26)		
When AD was completed^j^			
Before admission to TC^k^	22 (30)		
During admission to TC^k^	51 (70)		
Trial Arm			
Intervention*	53 (70)	63 (41)	116 (51)
Usual care*	23 (30)	89 (59)	112 (49)

^a^AD = Advance directive

^b^QoL = Quality of life

^c^SMMSE = Standardised Mini Mental State Examination (SMMSE)

^d^ GDS-15 = Geriatric Depression Scale. Due to missing data, *n* = 78 for AD completers and *n* = 147 for AD non-completers

^e^There was some missing data on the BMI and therefore *n* = 79 for AD completers and *n* = 140 for non-completers

^f^QoL = Quality of life. Due to missing data, *n* = 71 for AD completers and *n* = 137 for AD non-completers

^g^GPCP = Good palliative care plan

^h^EPG = Enduring power of guardianship

^i^MD = Medical documentation

^j^Some data missing on when the AD was completed were missing for *n* = 4 COACH participants

^k^TC = Transition care

*Kruskall Wallis test statistically significant at 0.05

## Results

Recruitment ended when the target recruitment level (determined through sample size calculation) for the COACH trial of 230 older adults, who were followed up to 12 months, was reached [43]. Two individuals were not registered for Medicare benefits and so were excluded from the analysis. Table 1 shows the descriptive results from the total sample (*n* = 228). At baseline, individuals had a mean age of 84.1 years and mean EQ-5D 3 L, Barthel and SMMSE scores of 0.46, 59.8 and 23.2, respectively. The baseline GDS-15 mean score was 4.8 while that for BMI was 24.3. The majority of participants in the study sample were female (*n* = 138, 61 %) and, at the end of the 12 months follow up period, the majority remained alive (*n* = 175, 77 %) while nearly half (95, 46 %) registered a QoL improvement according to the EQ-5D 3 L (mean EQ-5D 3 L improvement over 12 months of 0.33). Of the 23 % (*n* = 53) of individuals who had died at the end of the study, almost all died within a hospital (*n* = 31, 58 %) or in a residential aged care or respite facility (*n* = 19, 36 %). For some decedents, the place of death was also the first discharge destination from TC. For instance, the first discharge destination post-TC for 12 (39 %) of the decedents who died in hospital was readmission to hospital while 10 (53 %) of those who died in a residential aged care or respite facility were first discharged to a residential aged care facility (results not shown in Table 1). Of the total sample, 76 (33 %) completed ADs with the majority of these completing a GPCP (*n* = 35, 44 %) and almost equal numbers completing either an EPG (*n* = 21, 26 %) or an MD (*n* = 20, 25 %). Eighteen of the 53 decedents (34 %) had completed ADs (results are shown in Table 1).

Compared to those who did not complete ADs (mean scores in Table 1), individuals who did so were, on average, older (85.8 vs 83.3 years) and had slightly lower QoL and functional status (0.41 vs 0.49 and 57.5 vs 61.0 for EQ-5D 3 L and Barthel scores, respectively). The AD completers also had marginally lower SMMSE and BMI scores (22.2 vs 23.6, 24.0 vs 24.5, respectively), higher GDS-15 scores (5.8 vs 4.3) and were much more likely to be in the intervention group (70 % vs 30 %). The completion rate in the intervention group was higher than that in the usual care group (46 % vs 20 %). All differences were statistically significant except those for the Barthel, SMMSE and BMI scores. There was no statistically significant difference between those that completed ADs and those that did not in terms of gender, proportions that had registered QoL improvements or had died at the end of the study (12 months) or in relation to place of death. Though there was no statistically significant association between completing ADs and when these ADs were completed (i.e. before or during admission to a TC), results not presented in Table 1 showed that among AD completers, the time of completion differed statistically according to trial arm. In particular, 76 % of AD completers in the intervention arm completed these documents during admission to TC compared to 55 % in the control arm. As shown in Table 2, AD completers (defined according to type of AD completed) differed significantly by SMMSE score (lower scores being associated with completion of EPG) and by trial arm (majority of GPCP (86 %) and EPG (67 %) completers were in the intervention arm while majority of individuals who completed MDs were in the control arm). There were trends for individuals who completed an EPG to be female (*p* = 0.06) and younger (*p* = 0.09). There were no statistically significant relationships between the type of AD completed and any other variables.

**Table 2:** Type of advance care directive by participant characteristics

Variable	GPCP^a^	EPG^b^	MD^c^
	*n* = 35	*n* = 21	*n* = 20
	Median; mean (SD)	Median; mean (SD)	Median; mean (SD))
Age in years	86.0; 85.0 (7.5)	87.0; 84.8 (7.2)	87.5; 88.0 (5.6)
Baseline QoL^d^ (EQ-5D score)	0.33; 0.38 (0.33)	0.45; 0.46 (0.26)	0.38; 0.41 (0.33)
Baseline functional status (Barthel Index)	57.0; 55.5 (23.3)	61.0; 59.8 (19.2)	60.5; 58.4 (20.9)
Baseline cognitive function (SMMSE^e^ score)*	25.0; 23.3 (5.7)	19.5; 19.2 (5.8)	23.5; 23.4 (4.3)
Baseline depressive symptoms (GDS-15^f^ score)	6.0; 5.8 (2.7)	5.0; 5.6 (2.9)	4.5; 6.0 (3.6)
Body mass index (BMI)^g^	22.2; 23.6 (6.5)	24.0; 24.9 (5.3)	22.9; 23.9 (5.0)
Gender [n,(%) male]	15 (43)	12 (57)	5 (25)
n (%) who had a QoL^h^ gain over 12 months	13 (41)	7 (41)	8 (44)
n (%) who had died at the end of 12 months	10 (29)	6 (29)	2 (10)
Place of death [n (%) in each column]			
Hospital	6 (60)	3 (50)	1 (50)
Residential Care/Respite	4 (40)	3 (50)	1 (50)
Home/Independent living unit	0	0	0
When AD was completed^i^			
Before admission to TC^j^	0	17 (81)	5 (25)
During admission to TC^j^	35 (100)	2 (10)	15 (70)
Trial Arm			
Intervention*	30 (86)	14 (67)	9 (45)
Usual care*	5 (14)	7 (33)	11 (55)

^a^GPCP = Good palliative care plan

^b^EPG = Enduring power of guardianship

^c^MD = Medical documentation

^d^QoL = Quality of life

^e^SMMSE = Standardised Mini Mental State Examination

^f^GDS-15 = Geriatric Depression Scale. Due to missing data, *n* = 34 for GPCP, 20 for EPG and 18 for MD

^g^There was some missing data on the BMI: *n* = 34 for GPCP, = 21 for EPG & 20 for MD

^h^QoL = Quality of life. Because of missing data, *n* = 32 for GPCP, 17 for EPG and 18 for MD

^i^Some data on when the AD was completed were missing: *n* = 19 for EPG and *n* = 20 for MD

^j^ TC = Transition care

*Kruskall Wallis test statistically significant at 0.05

Table 3 shows the bivariate relationship between costs and a number of patient characteristics. Multiple imputations were used to account for missing data on the GDS-15 (1 %), BMI (4 %), EQ-5D 3 L (9 %), when ADs were completed (2 %) and costs (16 %). And as the results for the imputed data were similar to those based on unimputed data, we only show the former in the paper. The only costs that differed according to whether or not one had completed ADs (and type of AD completed) were costs of the COACH intervention – higher for those who completed ADs ($274 vs $149) and higher for individuals who completed GPCPs and EPGs in particular ($359 and $262, respectively vs $168 for MDs). In a sensitivity analysis, four individuals, considered to be outliers, were excluded due to extremely high total costs of over $30,000 per individual. The exclusion of these four reduced the difference in total health care utilisation costs between ADs completers and non-completers to approximately $15,000 per patient. In addition to total costs, the type of costs that differed significantly based on gender (higher for males) were those for hospitalisations, tests, treatments and investigations. Pharmaceutical costs for clients who recorded a QoL improvement at the end of 12 months were also significantly lower than those for individuals who recorded no change or QoL deterioration. Some costs also differed according to whether or not the patient had died at the end of 12 months. These were costs for drugs, hospitalisation, GP and other primary care visits (other costs) and total costs. In general, decedents exhibited higher total and hospitalisation costs but lower pharmaceutical and other costs.

**Table 3:** Costs by advance care directive completions and selected demographic characteristics (Australian dollars)

	Type of costs: figures are mean (Standard deviation)						
Variable	Drugs	Hospitalisation	Tests, treatments & Investigations	Consultations	Intervention	Other^a^	Total
	*n* = 228	*n* = 228	*n* = 228	*n* = 228	*n* = 228	*n* = 228	*n* = 228
Was an AD^b^ completed?							
Yes (*n* = 76)	4,605(4,949)	33,534(63,669)	3,104(4,109)	2,323(1,957)	285(212)*	172(267)	43,955(64,490)
No (*n* = 152)	5,648(5,794)	44,396(86,314)	3,793(6,249)	2,531(2,353)	153(210)*	181(262)	56,910(90,740)
Type of AD^b^ completed							
GPCP^c^ (*n* = 35)	4,745(5,393)	30,256(44,876)	2,162(2,571)	1,843(1,694)	356(172)*	161(271)	39,423(43,911)
EPG^d^ (*n* = 21)	4,867(5,866)	46,034(91,302)	3,749(5,445)	2,589(1,842)	277(228)*	159(289)	57,610(92,844)
MD^e^ (*n* = 20)	4,085(2,834)	26,147(58,077)	4,076(4,529)	2,883(2,363)	172(218)*	206(248)	37,548(60,049)
Gender							
Females (*n* = 138)	5,313(5,419)	32,305(72,418)*	2,873(3,688)	2,378(2,158)	196(218)	191(254)	43,485(74,557)*
Males (*n* = 90)	5,281(5,747)	53,763(88,139)*	4,622(7,611)	2,590(2,335)	199(222)	158(278)	66,557(93,207)*
Did individual have a QoL gain?							
Yes (*n* =105)	5,242(5,866)	34,530(81,028)	4,448(7,253)	2,420(2,161)	206(221)	211(300)	47,027(87,102)
No (*n* = 123)	5,237(5,430)	40,390(60,029)	2,932(4,188)	2,547(2,387)	193(218)	151(231)	51,718(62,788)
Was individual dead at 12 months?							
Yes (*n* = 53)	4,698(7,024)*	63,139(100,000)*	3,985(7,107)	2,379(2,656)	181(208)	106(210)*	74,439(110,000)*
No (*n* = 175)	5,483(5,012)*	34,002(70,232)*	3,436(5,112)	2,487(2,088)	202(223)	200(274)*	45,976(71,992)*
Place of death							
Hospital (*n* =31)	4,351(7,099)	87,896(130,000)	5,034(8,793)	2,637(3,233)	223(213)	59(112)	100,000(140,000)
Residential care (*n* = 19)	3,931(3,109)	28,812(25,740)	1,675(1,338)	1,912(1,487)	142(197)	190(307)	36,610(27,035)
Home/Ind. living unit (*n* = 3)	13,141(18,044)	24,710(42,798)	7,763(6,959)	2,679(1,847)		51(88)	48,343(67,221)
Total sample	5,300(5,538)	40,775(79,507)	4,083 (5,951)	2,786 (2,256)	188 (219)	201 (279)	55,214 (88.421)

*Kruskall Wallis test statistically significant at 0.05; ^a^Other = primary care and general practice; ^b^AD = Advance directive; ^c^GPCP = Good palliative care plan^; d^EPG = Enduring power of guardianship; ^e^MD = Medical documentation

Table 4 reports the results of a series of the two-step endogenous treatment-regressions are based on multiply imputed data as well as on data that were adjusted for censoring. The results of the first step show that the probability of completing ADs increased as people aged, experienced a rise in depressive symptoms (as measured by the GDS-15) and was also higher among females compared to males. The results from the second step indicate that being older was associated with lower healthcare costs for all type of costs except for ‘GP and primary care visit costs’ that were higher for older people. Further, a higher BMI was predictive of higher pharmaceutical costs while a higher SMMSE and having recorded a QoL gain at 12 months were associated with higher costs for tests, treatments and investigations. A higher SMMSE score was also indicative of higher costs for consultations. Lastly, decedents at 12 months were associated with higher GP and primary care visit costs. Data on four clients who withdrew from the study were censored. The regression model with multiple imputed data and adjustment for censoring showed, as above, a negative relationship between total costs and age but also showed that higher total costs were associated with being a decedent at 12 months. No statistically significant relationship was seen between completing ADs and any of the costs in all the models above.

**Table 4:** Predictors of completing Advance Directives and Costs (based on binary and linear model regressions of multiply imputed data)

		Dependent variables for 2^nd^ step of the regression analyses are different types of costs: figures are coefficient (Std. Err.)						
	Independent variable	Drugs	Hospitalisation	Tests, treatments & Investigations	Consultations	Other^a^	Total	Total (adjusted for censoring)^b^
		*n* = 228	*n* = 228	*n* = 228	*n* = 228	*n* = 228	*n* = 228	*n* = 228
Predictors of Costs								
	Was an AD^c^ completed? (1 = yes; 0 = No)	0.02 (0.24)	0.93 (0.58)	0.47 (0.32)	0.22 (0.23)	0.96 (0.51)	0.64 (0.42)	0.19 (0.11)
	Gender(1 = Female, 0 = Male)	0.05 (0.13)	−0.20 (0.33)	−0.22 (0.17)	−0.06 (0.12)	−0.29 (0.29)	−0.17 (0.24)	−0.05 (0.06)
	Age (in years)	−0.02 (0.01)*	−0.09 (0.03)*	−0.06 (0.01)*	−0.02 (0.01)*	0.10 (0.03)*	−0.06 (0.02)*	−0.01 (0.00)*
	Baseline EQ-5D 3 L score	−0.15 (0.29)	0.44 (0.68)	0.44 (0.40)	0.00 (0.29)	0.27 (0.55)	0.30 (0.51)	−0.05 (0.13)
	Baseline Barthel Index	0.00 (0.00)	−0.01 (0.01)	−0.01 (0.01)	0.00 (0.00)	0.01 (0.01)	−0.01 (0.01)	0.00 (0.00)
	Baseline SMMSE^d^ score	0.02 (0.01)	0.00 (0.03)	0.04 (0.02) *	0.03 (0.01) *	0.03 (0.03)	0.01 (0.02)	0.01 (0.01)
	Baseline GDS-15^e^ score	0.00 (0.02)	−0.08 (0.06)	0.01 (0.03)	0.01 (0.02)	−0.03 (0.05)	−0.06 (0.04)	−0.01 (0.01)
	Body mass index	0.04 (0.01)*	−0.06 (0.03)	−0.01 (0.02)	0.01 (0.01)	0.03 (0.03)	−0.03 (0.02)	0.00 (0.01)
	Did individual have a QoL^f^ gain at 12 months? (1 = ‘Yes’, 0 otherwise)	−0.11 (0.15)	−0.20 (0.37)	0.44 (0.21) *	−0.03 (0.15)	0.48 (0.28)	−0.21 (0.27)	−0.23 (0.17)
	Dead at 12 months (1 = Yes, 0 = No)	−0.19 (0.16)	0.20 (0.39)	−0.05 (0.22)	−0.16 (0.15)	0.74 (0.32) *	0.09 (0.29)	0.29 (0.08) *
	Constant	8.98 (1.15)*	20.10 (3.15)*	13.1 (1.48)*	8.78 (1.01)*	−4.99 (2.73)	17.13 (2.04) *	11.89 (0.47)*
	R-Squared	0.19	0.09	0.18	0.09	0.08	0.10	0.25
		The dependent variable for each type of cost in the 1^st^ step of the regression analyses is whether or not an individual completed an Advance Directive: figures are coefficient (Std. Err.)						
		Drugs	Hospitalisation	Tests, treatments & Investigations	Consultations	Other^a^	Total	Total (adjusted for censoring)^b^
		*n* = 228	*n* = 228	*n* = 228	*n* = 228	*n* = 228	*n* = 228	*n* = 228
Predictors of AD completion								
	Gender (1 = Female, 0 = Male)	0.12 (0.04)*	0.10 (0.03)*	0.12 (0.04)*	0.17 (0.05)*	0.12 (0.03)*	0.10 (0.03)*	0.12 (0.03)*
	Age (in years)	0.03 (0.01)*	0.03 (0.01)*	0.03 (0.01)*	0.04 (0.01)*	0.03 (0.01)*	0.03 (0.01)*	0.04 (0.01*)
	Baseline SMMSE^d^ score	−0.02 (0.02)	−0.03 (0.02)	−0.03 (0.02)	−0.02 (0.02)	−0.02 (0.02)	−0.03 (0.02)	−0.03 (0.02)
	Baseline GDS-15^e^ score	0.08 (0.03)*	0.09 (0.03)*	0.09 (0.03)*	0.09 (0.03)*	0.12 (0.03)*	0.09 (0.03)*	0.09 (0.03)*
	constant	−2.92 (1.14)	−2.88 (1.16)	−2.66 (1.18)	−3.02 (1.19)	−2.94 (1.23)	−2.97 (1.15)	−2.98 (1.15)
	Likelihood-ratio test of independent equations	*χ* ^2^ = 18.60	*χ* ^2^ = 34.24	*χ* ^2^ = 46.01	*χ* ^2^ = 26.12	*χ* ^2^ = 9.93	*χ* ^2^ = 37.21	*χ* ^2^ = 38.60
		(*p* < 0.01)	(*p* < 0.01)	(*p* < 0.01)	(*p* < 0.01)	(*p* < 0.01)	(*p* < 0.01)	(*p* < 0.01)

^a^Other = primary care and general practice; ^b^Total costs calculated after adjusting for censoring using Lin’s method [64]; ^c^AD = Advance directive; ^d^SMMSE = Standardised Mini Mental State Examination; ^e^GDS-15 = Geriatric Depression Scale; ^f^QoL = Quality of life; *Statistically significant at 0.05 level

## Discussion

Our sample was made up of older people in TC, a type of intermediate care, at risk of readmission to hospital and death within the following 12 months. Overall, older people who received the coaching intervention in our study had statistically higher costs associated with completing ADs. Significantly and when compared to the control, the intervention group had a higher rate of AD completion as well as heavier staff involvement, which may have contributed to the higher costs in the latter. There was no statistically significant relationship between any other healthcare costs and completion of ADs regardless of whether or not the effect of other factors was controlled for. This result seems to be congruent with other studies that suggest that the use of ADs does not necessarily lead to significant cost savings [27, 28, 30, 31, 33–35] or to patients calling for more of the expensive, high technology treatment often used in terminal phases of illness [34]. The National Framework for Advance Care Directives recommends making ADs a routine part of patient contact with practitioners in health and aged care [17] and this result suggests it may be possible to do this in a cost neutral manner with the focus mainly on improving the end of life for older frail people rather than on managing costs.

The AD completion rate of 33 % in our entire sample was lower than that reported in a systematic review of interventions that promote ADs among older adults internationally (45.6 %) [66] but the rate in our intervention group (46 %) was comparable suggesting that the COACH intervention was as effective as similar international interventions designed to promote AD completion rates. [66] [66] As expected, there was a higher rate of completion for the intervention compared to usual care within the COACH study (46 % vs 20 %). The reason for this higher rate in the former was that the intervention encouraged older adults to consider their preferences for end of life care and to discuss these with their family and consider AD completion. This was in contrast to usual care in which these discussions were triggered when a participant was identified as palliative. Consequently, a higher proportion of individuals in the intervention arm last completed an AD during, rather than before, admission to the TC site. It is not surprising that the majority of ADs completed were GPCPs as this was the main ‘prompt’ document in use at the TC site. In addition, a comparable South Australian study documenting end of life decisions in residential care facilities found a similar result [67]. Our study also showed that 34 % of decedents had completed ADs, which is comparable to results from similar studies that report the proportion of deaths preceded by completion of any end of life document range between 23 % (Italy) and 51 % (Switzerland) [68]. As also noted by other Australian studies [14], almost all individuals in our sample died in a hospital or another institutional setting which is at odds with people’s preferences as previously reported [14]. Further, for some decedents, the place of death was also the first discharge destination after TC and it is unclear to what extent decedents’ first discharge destinations and places of death represented their preferences. As data on preferred place of death was not collected in our study, it was not possible to empirically test whether place of death reflected patients’ preferences. Further research is required before strong conclusions can be drawn on these findings.

The National Framework for Advance Care Directives in Australia [17] reports that of people considering ADs, 10 % are near death, 30 % are chronically ill and 60 % are well which suggests that most individuals completing ADs are not experiencing major illness or disability. Contrary to these findings, older people who completed ADs in our study had lower QoL than older people generally. It is important to recognise however that our study was completed in a residential TC setting and study participants had a recent major illness or injury, and significant disability that put them at risk of not returning to live in the community. These study participants were thus are not representative of the general older Australian population. Completion of an EPG was associated with lower SMMSE scores indicating that individuals with greater cognitive impairment had a higher probability of having SDMs.

The higher overall and component (hospitalisations, tests, treatments and investigations) costs for males compared to females may be explained by the longer in-hospital length of stay for the former group (five days longer on average). Total costs for decedents (driven in a large part by hospitalisation costs) were higher (nearly twice as high) than those for survivors and these results were also borne out by those from international studies: a study reported that costs for US decedents in 2004 were over six times higher than those for survivors [69] while the figure was between 9.4 and 13.3 in Denmark [39] and 13.5 in the Netherlands [70].

The use of endogenous treatment-regression models showed no statistically significant relationship between completing ADs and costs, whilst controlling for the joint effect of other variables. The regression results also show that when costs were split up into different cost components, none of these costs showed a statistically significant relationship with competing ADs. In line with previous research [19, 40–42], there was some evidence from our study suggesting that the decision to complete ADs was influenced by age, depressive symptoms and gender. This therefore implied that treating this decision as an endogenous effect in our models was appropriate. Though some research suggests that ADs could reduce inappropriate over-investigation and over-treatment of patients, leading to significant cost savings for the health system [26, 29, 32, 36–38], our results suggest that completion of ADs did not impact the costs of healthcare provision but were cost neutral. This lack of association between costs and completion of ADs has however been shown in a few other studies [27, 28, 30, 31, 33–35] and could be due to a number of factors. Firstly, a recent systematic review [71] has shown that the effects of ADs seem to be related to an increased frequency of out-of-hospital care aimed at increasing the patient’s comfort instead of prolonging their life. Compared with in-hospital care, this type of care is relatively inexpensive and hence does not affect the overall healthcare costs significantly. Similar to this review, our study indicated that the only costs that were higher for AD completers, in comparison to AD non-completers, were consultation costs (though this result was statistically insignificant) the majority of which are for out-of-hospital visits. Secondly, there is a view that clinical uncertainty at the end of life may mean that resource utilisation is unchanged until prognosis is clearer and physicians, patients and SDMs are certain about either going ahead with expensive treatment or withdrawing it [34]. This means that until such time, costs between AD completers and non-completers are unlikely to be significantly different. It is also possible that the structural barriers in access to care identified as characterising the delivery of end of life care [72] may mean that completing ADs does not readily lead to substantial cost movements as healthcare service utilisation does not change substantially. Such barriers could include cultural or ethnic norms as well as limited access to care for geographic or financial reasons [72]. Further, ADs can sometimes be too specific to account for significant changes in medical treatment and associated healthcare costs [17, 73]. Lastly, our result may also be reflective of the lack of fuller integration of AD policies and procedures within the South Australian health system needed to make the system more responsive and ensure that AD implementation occurs at both the micro and macro level rather than at just the former. Results from the SUPPORT trial found that efforts to improve end of life decision making need to focus on greater individual and societal approaches if established practices are to be changed [74, 75]. Hickman et al. [76] also suggest that for “…advance directives to be effective, they need to be integrated into each part of the system of care, including emergency medical service protocols and regulations.”

This study had some limitations. The total follow up period of 12 months was relatively short so the longer-term relationship between ADs, costs and other variables is not known. In addition, we did not have information on whether medical care after completion of ADs was consistent with the preferences expressed within these ADs, which would have given a much more accurate picture of healthcare utilisation and associated costs. A longer follow-up period and/or model-based extrapolation would be helpful in this regard and should be considered in future research. There was a proportion of missing data relating to resource use and other patient variables. However, robust statistical imputation methods were employed to deal with these missing data and the results from the imputed data and complete case analysis are broadly similar. Costs for residential aged care and other support services for this population are likely to be substantial but these were not included as robust data on these costs were not collected as part of the trial. There is also large variation around the mean estimates, particularly relating to the cost data, which is to be expected because this study has a relatively small sample size. Consequently, a larger cohort is needed to substantiate these preliminary findings. Finally, Advance Care Planning (ACP) has been gaining prominence in Australia due to a recent shift from documentation towards the process of ACP [77]. Advance Directives are only a small part of ACP and therefore future research should consider exploring the relationship between healthcare costs and ACP to gain a much broader understanding of how decisions about end of life care impact on healthcare costs.

## Conclusion

This study has documented, and confirmed, the substantial pharmaceutical, hospital and other costs associated with caring for older people nearing the end of life. However no significant association was found between health care utilisation costs and ADs. If one of the rationales for the introduction of ADs is improving the end of life experience for older people and their families whilst being cost neutral with regards to healthcare expenditures on older people, then this study provides some evidence to support this proposition. However, further research is needed to substantiate these findings in larger and more diverse clinical cohorts of frail older people.
